# Development and evaluation of a multiplex PCR-based dual-platform targeted sequencing framework for precise differentiation of lumpy skin disease virus

**DOI:** 10.3389/fmicb.2026.1916665

**Published:** 2026-08-25

**Authors:** Yuqi Wang, Yifei Wang, Zilong Bai, Suqiu Wang, Lin Wang, Wei Zhang, Zhiqiang Gao, Jing Pu, Sile Du, Congliang Deng, Liwei Xu, Wangxue Wu, Xiangpeng Zhao, Tong Ren, Dan Wu, Jiawei Zhang, Qiang Gu, Shaolin Wang, Xiju Shi

**Affiliations:** 1 State Key Laboratory of Veterinary Public Health and Safety, College of Veterinary Medicine, China Agricultural University, Beijing, China; 2 Science and Technology Research Center of China Customs, Beijing, China

**Keywords:** amplicon sequencing, Capripoxvirus differentiation, long-read sequencing, lumpy skin disease virus, multiplex PCR, short-read sequencing

## Abstract

**Background:**

Lumpy skin disease virus (LSDV) shares over 96% genomic identity with goatpox and sheeppox viruses, presenting severe diagnostic challenges due to cross-reactivity.

**Methods:**

To address this bottleneck, we established a targeted sequencing framework integrating multiplex PCR with short-read and long-read platforms. By sequentially screening target pathogens, identifying low-homology genes, and designing short and gradient long-fragment primer pools, we evaluated these dual-platform panels using highly homologous poxvirus samples.

**Results:**

The short-read panel stably detected target viruses at inputs as low as 5.26 ×10^1^ copies/μL. Under strict alignment criteria, LSDV mapping rates reached 42.91%, suppressing non-target signals to 3.05%. The Nanopore-Targeted Sequencing (NTS) long-amplicon strategy successfully eliminated homologous interference. By applying length-dependent diagnostic thresholds (≥ 100 reads for short amplicons; ≥ 50 reads for long amplicons), precise species-level identification was achieved, maintaining near-zero cross-reads (0–5) in ultra-long regions. Crucially, the field-deployable NTS workflow enabled complete detection in approximately 4 h.

**Conclusion:**

This complementary strategy seamlessly meets both laboratory demands for high-sensitivity enrichment and frontline requirements for rapid typing, providing a reliable tool for LSDV surveillance, mutation tracking, and outbreak control.

## 1 Introduction

Lumpy skin disease (LSD) is an important infectious disease of animals caused by the lumpy skin disease virus (LSDV). LSDV belongs to the genus Capripoxvirus (CaPVs) within the family Poxviridae. It is a double-stranded DNA virus with a genome of approximately 150 kb encoding 156 open reading frames (ORFs) (Tulman et al., 2002; Babiuk et al., 2008). The disease is primarily characterized by fever, skin nodules, enlarged lymph nodes, and a sharp drop in milk production, causing severe economic losses to the animal husbandry industry (Moudgil et al., 2024).

Lumpy skin disease virus was first reported in Zambia in 1929 and subsequently spread to Africa, the Middle East, and Europe (Mazloum et al., 2023; Sendow et al., 2024). In 2019, it was introduced into Xinjiang, China, and rapidly spread to multiple provinces across the country (Flannery et al., 2022). LSDV shares over 96% genomic homology with goatpox virus (GTPV) and sheeppox virus (SPPV) of the same genus, leading to serological cross-reactivity and cross-immune protection. This makes the accurate differentiation of the three a technical challenge in disease prevention and control (Tuppurainen and Oura, 2012). The virus is primarily transmitted mechanically by blood-feeding insects (e.g., mosquitoes and flies) and can also spread through direct contact or contaminated environments (Sohier et al., 2019). Its incubation period under natural infection can reach 28 days, further increasing the difficulty of prevention and control (Hamdi et al., 2021; Sudhakar et al., 2023).

Currently, LSDV detection methods mainly include virus isolation, serological testing, and molecular biology detection technologies. Although virus isolation is considered the “gold standard” for pathogen identification, its long operation cycle and high biosafety requirements make it unsuitable for rapid on-site detection. Serological methods, such as the virus neutralization test (VNT) and enzyme-linked immunosorbent assay (ELISA), can be used for population infection background investigations, but their main limitation is the inability to distinguish between LSDV, GTPV, and SPPV, coupled with low antibody sensitivity in the early stages of infection, which is unconducive to early epidemic interception (Babiuk et al., 2008; Zeedan et al., 2019; Selim et al., 2021). Molecular detection technologies such as conventional PCR and qPCR have improved sensitivity and specificity. For example, a TaqMan MGB qPCR method established in one study reached a limit of detection (LOD) of 8 copies/μL, but issues such as a single target, short amplicon length, and the inability to fully resolve highly homologous genomic differences remain (Bowden et al., 2008; Das et al., 2012). Although isothermal amplification technologies (e.g., LAMP, RPA) are suitable for rapid on-site screening, result interpretation is susceptible to subjective factors, and their differentiation ability is limited (Vidanović et al., 2021; Lin et al., 2022; Cao et al., 2023).

In recent years, the emergence of high-throughput sequencing technologies has provided a novel approach to overcoming the aforementioned technical bottlenecks. Short-read sequencing has been widely applied in pathogen genomic analysis due to its high throughput and cost-effectiveness. While short-read sequencing provides massive overall depth, the physical length of a single continuous read (e.g., 150 bp) is fundamentally limited. Therefore, it cannot capture multiple distant SNPs or resolve complex structural variations (Indels) within the same continuous molecular phase (haplotype block), hindering precise haplotype phasing and species discrimination in highly homologous viral genomes (Guan et al., 2016; Jiang et al., 2022; Tan et al., 2024; Xu et al., 2026). In contrast, Nanopore sequencing offers significant advantages including long read lengths, real-time analysis, and high portability. By capturing multiple consecutive variable loci in a single read, Nanopore sequencing provides richer SNP information, thereby substantially enhancing the discriminative power for highly homologous viruses. This makes it particularly suitable for rapid, on-site pathogen tracing during outbreak scenarios (Theuns et al., 2018; Charalampous et al., 2019; Athanasopoulou et al., 2021; Wang et al., 2021; Huggins et al., 2022). Nevertheless, direct metagenomic sequencing continues to face challenges when analyzing samples with low viral loads or severe host background interference (Yin et al., 2021; Diao et al., 2021). As a targeted enrichment strategy, targeted amplicon sequencing specifically amplifies key discriminatory regions. When coupled with nanopore platforms, this approach has formalized into Nanopore-Targeted Sequencing (NTS). NTS perfectly integrates the robust enrichment capabilities of multiplex PCR with the long-read and real-time advantages of nanopore technology, enabling highly sensitive and specific viral genotyping even within complex samples (Mamanova et al., 2010; Yin et al., 2024; Liang et al., 2025). While this technology has been successfully applied to the detection of pathogens such as avian influenza virus (AIV), a systematic targeted sequencing assay has not yet been established for LSDV detection (Croville et al., 2024).

Addressing the current differentiation challenges of LSDV in clinical diagnosis and epidemiological investigations—particularly the high genomic homology among LSDV, GTPV, and SPPV, which causes traditional molecular and serological detection technologies to be limited by cross-reactivity or insufficient resolution—a critical diagnostic gap remains. Conversely, as previously discussed, un-enriched whole-genome sequencing (WGS) generates massive redundant data and is inefficient for routine species-level screening. Although general amplicon and nanopore sequencing are widely used in pathogen detection, a systematic targeted sequencing framework designed to overcome the > 96% genomic homology barrier for precise Capripoxvirus differentiation has not yet been fully established. To address this, this study developed a dual-platform targeted sequencing framework deeply integrating multiplex PCR targeted amplification with high-throughput sequencing technologies. By designing multiplex PCR primer libraries specifically targeting the variable and key functional regions of the LSDV genome, we aimed to effectively enrich viral nucleic acids in complex background samples. As a core technical approach, this study introduced a complementary strategy integrating both short-read targeted sequencing and long-read NTS. By systematically evaluating the dynamic interplay among amplicon length, amplification efficiency, and inter-species cross-reactivity using reference nucleic acid samples, we established precise bioinformatic interpretation thresholds. Ultimately, this approach provides a reliable molecular strategy to circumvent the cross-reactivity issues among capripoxviruses, providing a scalable and standardized methodological framework for future diagnostic development, ranging from broad-spectrum farm surveillance to stringent cross-border quarantine typing.

## 2 Materials and methods

### 2.1 Sample sources and viral nucleic acid extraction

The nucleic acids of LSDV, GTPV, and SPPV strains used in this study were provided and identified by the Science and Technology Research Center of China Customs. Specifically, the initial identities of the LSDV, GTPV, and SPPV samples were pre-evaluated using the standard quantitative real-time PCR (qPCR) assay recommended by the Chinese national standard (GB/T 39602–2020, GB/T 45105–2024). To evaluate assay performance in complex biological backgrounds and address clinical applicability, authentic clinical positive specimens—specifically two lesion swabs designated as Clinical Sample 1 (4.27 × 10^4^ copies/μL) and Clinical Sample 2 (1.49 × 10^4^ copies/μL), as determined by absolute quantitative qPCR—and confirmed uninfected ruminant blood and tissue samples (utilized as clinical negative controls, CN, and background matrices) were additionally collected.

All nucleic acid samples were extracted using TIANamp Virus DNA/RNA Kit (TIANGEN). The extracted DNA was measured for concentration and purity using a Qubit 4.0 fluorometer and a NanoDrop spectrophotometer (Thermo Fisher Scientific, United States) and then stored at −80 °C for subsequent primer validation and library construction sequencing.

### 2.2 Multiplex targeted primer library design

To achieve the dual goals of “high-sensitivity screening in the laboratory” and “rapid, high-specificity identification at the customs frontline,” this study specifically designed and screened specific primer libraries for the Capripoxvirus (CaPVs) genome using a complementary short-read and long-read strategy. All primers were designed based on multiple sequence alignments of the reference genomes of LSDV, GTPV, and SPPV in the NCBI database (accession numbers: AF325528.1, NC_004003.1, and NC_004002.1). To adapt to the read length characteristics of different sequencing platforms, the primer sets were divided into a “Short-read sequencing primer set” and a “Long-read sequencing primer set” as follows:

#### 2.2.1 Short-read amplicon primer design

Short-read targeted sequencing aimed to utilize the high-throughput advantages of the MGI platform to achieve robust target enrichment and reliable detection even at low nucleic acid inputs in laboratory environments. The LSDV reference sequence was aligned with the SPPV and GTPV genomes using full-length multiple sequence alignment (MSA) performed with MAFFT (version 7.453) (Katoh and Standley, 2013) to evaluate the sequence similarity among the three pathogens. Genomic regions with low homology were prioritized, from which highly specific fragments and segments with clear gene function annotations were extracted as the candidate target pool. For the target sequences in this pool, 2 to 4 target sites were assigned to each region. Centered around these sites, the expected amplicon length was strictly set to 100–200 bp using the ATOPlex customized primer design platform to define the amplification boundaries. To ensure the specificity and discriminatory power of the system, all initially designed candidate primers and their corresponding amplicon nucleic acid sequences were subjected to rigorous BLASTn alignment validation using NCBI BLAST+ (version 2.9.0) (Camacho et al., 2009) with strict parameters (*E*-value threshold of 1e-5, word size of 11, and minimum query coverage of 97%). Screening criteria required that the primer binding region sequences must be highly conserved within the CaPVs genus to ensure that all three pathogens could be effectively amplified. Meanwhile, the internal nucleic acid sequence homology of the amplicons among the three pathogens must be less than 97%, with no cross-homology with non-target background microorganisms. It should be specifically noted that, relying on MGI’s multiplex primer evaluation algorithm, non-specific thermodynamic interference factors such as primer dimers and internal hairpin structures were fully considered and automatically eliminated during the calculation phase. The finally screened primers were directly submitted to the MGI platform for industrialized synthesis and purification in the form of a customized Multiplex Amplification Panel.

#### 2.2.2 Long-read amplicon primer design

Unlike the short-read strategy based on specific functional annotation regions, the primer sets designed for long-read sequencing were mainly designed for rapid and accurate identification in frontline scenarios such as customs quarantine. To systematically find the most species-specific differentiating regions, this study used the PrimalScheme software (version 1.3.2) for unbiased target region screening (Kent et al., 2024). First, using the LSDV genome (AF325528.1) as a single core template, multiple gradients of expected amplicon lengths (approximately 500–600 bp, 800–900 bp, 1,000–1,100 bp, 1,500 bp, and 2000 bp) were directly set at the whole-genome level using the primer design tool PrimalScheme to generate an initial long primer set covering the entire genome. All generated candidate primers and their corresponding amplicon sequences were strictly aligned in parallel with the SPPV and GTPV genomes using the Blastn software. The screening criteria were set as follows: the primer sequences must be highly conserved to simultaneously anchor the three pathogens, but the internal sequence identity of their amplicons among the three pathogens must be <97%. This strict homology threshold excluded highly conserved regions, ensuring that the remaining candidate fragments contained a very high density of species-specific single nucleotide polymorphisms (SNPs) and structural variants (Indels). In addition, to evaluate broad geographical applicability, an *in silico* alignment of our primer sets was conducted against representative global LSDV whole-genome sequences retrieved from GenBank, encompassing recent African, European, and Asian outbreak strains.

### 2.3 *In vitro* validation of the NTS long fragment primer library

For the NTS long fragment primer library, *in vitro* validation was conducted to verify the amplification specificity of each candidate primer pair. Specifically, singleplex PCR was performed using LSDV, GTPV, and SPPV genomic DNA as templates. Subsequently, a no-template control (NTC) system was established to evaluate and exclude non-specific interference. Based on the in vitro screening results, primers meeting the criteria were combined into multiple independent long-fragment multiplex PCR reaction groups according to their expected amplification lengths and thermodynamic parameters. For the extracted nucleic acids, the multiplex amplification reactions were performed independently on a ProFlex™ PCR system. The 25 μL reaction system contained: 12.5 μL 2 × Multiplex PCR Kit (Vazyme), pre-mixed specific multiplex primer sets (final concentration of each primer: 0.2 μM), and 2 μL of nucleic acid template. The thermal cycling program was performed as follows: an initial denaturation at 95 °C for 5 min; followed by 30 amplification cycles consisting of denaturation at 95 °C for 15 s, annealing at 60 °C for 15 s, and extension at 72 °C for 20 s; and a final extension at 72 °C for 5 min. For reactions with expected targets > 1,500 bp, the extension time per cycle was increased to 1–2 min. After the amplification products were quality-checked by 1.5% agarose gel electrophoresis, they were purified using VAHTS DNA Clean Beads (Vazyme) to serve as the starting targeted templates for subsequent long-read sequencing library construction.

### 2.4 Short-read platform-based targeted sequencing and workflow

Unlike the long-read NTS two-step method, the 97-plex targeted primers for short amplicons were directly integrated into the MGI library construction workflow without preliminary *in vitro* gel electrophoresis validation. For such a large-scale multiplex pool, evaluating primer subsets via traditional gel testing fails to accurately predict the competitive amplification dynamics and primer interactions within the final complex mixture. Instead, assay specificity and signal integrity were ensured through a three-tier approach: (1) rigorous *in silico* optimization of thermodynamic parameters to minimize dimer formation using the MGI ATOPlex web-based customized panel design platform; (2) stringent magnetic bead purification (AMPure XP) during library preparation to physically eliminate unreacted primers and short dimers (<100 bp); and (3) highly specific bioinformatic filtration, where diagnostic confirmation relies on exact sequence matching (>120 bp, 100% identity) rather than mere fragment size.

The LSDV nucleic acid samples were quantified and diluted into three concentration gradients of 5.26 × 10^3^, 5.26 × 10^2^, and 5.26 × 10^1^ copies/μL based on standard curve calculations. Simultaneously, four replicates each of GTPV nucleic acid samples and a negative control were set up. During library construction, 10 μL of each sample was used. Two rounds of multiplex targeted amplification, purification, and sequencing adapter ligation were performed using the customized ATOPlex kit to obtain the final library. After the purified and enriched sequencing libraries passed quality inspection, DNA nanoballs (DNBs) were prepared using rolling circle amplification (RCA) technology and loaded onto the MGISEQ-200 platform for PE100 paired-end sequencing.

### 2.5 Long-read platform-based targeted sequencing and workflow

To demonstrate cross-platform compatibility for long-read targeted sequencing, multiplex PCR amplicons derived from nucleic acids extracted from initial positive samples were processed in parallel on both Oxford Nanopore Technologies (ONT) and MGI (CycloneSEQ) platforms. For ONT library preparation, 80 ng of purified product was used as input with the ONT Rapid Barcoding Kit (SQK-RBK114) for transposase cleavage and adapter ligation, and loaded onto a MinION R10.4.1 Flow Cell. Total turnaround time (encompassing extraction, multiplex amplification, and data acquisition) was recorded to assess rapid field-detection feasibility. In parallel, to verify cross-platform applicability, 300 ng of purified product from the same batch was processed on the MGI CycloneSEQ long-read platform using the CycloneSEQ 24 barcode library preparation kit according to manufacturer instructions.

To further evaluate the analytical performance, diagnostic thresholds, and limit of detection (LoD) in complex biological backgrounds, three representative primer sets generating varying amplicon lengths (P1, P3, and P7) were selected for follow-up matrix spike-in and clinical sample evaluations using the ONT sequencing workflow. A serial dilution gradient of LSDV nucleic acid (10^1^, 10^2^, and 10^3^ copies/μL) was spiked directly into nucleic acid extracts derived from confirmed uninfected ruminant blood and tissue samples (clinical negative controls, CN). These spiked matrix samples, along with authentic clinical positive specimens (Clinical Sample1 and Clinical Sample2) and CN controls, were amplified using the selected P1, P3, and P7 primer sets, followed by ONT library construction and sequencing to quantify target coverage and off-target background noise.

### 2.6 Bioinformatics analysis and diagnostic interpretation criteria

For the output data from different sequencing platforms, differentiated bioinformatics processing and sequence alignment strategies were adopted, with all analysis software versions and citations detailed below.

For the MGI platform’s short-read raw data, quality control and adapter trimming were performed using fastp (version 0.23.4) (Chen et al., 2018) to obtain high-quality clean reads. Subsequently, paired-end read assembly was executed using PEAR (version 0.9.6) (Zhang et al., 2014). To achieve precise species-level identification and replace conventional slower BLAST searches with high-throughput efficiency, pblat (version 36) (Kent, 2002) was employed to align the assembled reads to the designed LSDV target regions, with strict alignment parameters set at 100% sequence identity and assembled read length > 120 bp.

For NTS long-read data, raw reads were first aligned to the LSDV target regions using Minimap2 (version 2.28-r1209) (Li, 2018) to extract mapped reads. The pblat software was then utilized for strict secondary screening, with rigorous filter parameters set to 98% sequence identity and a minimum alignment length of 250 bp or 400 bp (varying thresholds depending on the length of the designed amplicon). To evaluate species specificity and quantify off-target background noise, mapped reads were cross-aligned against reference genomes of GTPV (NC_004003.1) and SPPV (NC_004002.1). The Samtools toolkit (version 1.10–3) (Danecek et al., 2021) was used to calculate mapping reads and sequencing depth for each target region. Enrichment efficiency was defined as the percentage of reads aligned to the target viral region among total effective reads. Data visualization was accomplished using R software (version 4.3.0) with the ggplot2 extension package (version 3.4.2) (Wickham, 2016).

To ensure the reproducibility and standardization of the targeted sequencing method, preliminary diagnostic decision rules were established guided by the absolute read count distributions and empirical background noise observed during the analytical evaluation. A sample must first pass the aforementioned strict bioinformatics filtering thresholds. For the short-read sequencing panel, an individual target is considered valid if it achieves ≥ 50 mapped reads and a median sequencing depth of ≥50×. Detection of at least one valid species-specific target classifies the sample as species-positive (LSDV or GTPV).

For the NTS panel, length-dependent positivity thresholds were set at ≥100 mapped reads for short amplicons (P1–P4) and ≥50 for ultra-long amplicons (P5–P7) to balance fragment length and amplification efficiency. To suppress false positives from high-titer off-target viruses (GTPV/SPPV) and absorb run-to-run variations, these static cutoffs are supported by multi-layered safeguards: primer design enforcing <97% amplicon homology, stringent bioinformatic filtering (98% identity over ≥250/400 bp), and ultra-long amplicons acting as intrinsic run controls (yielding ≤5 off-target reads, obviating live parallel controls). Samples are categorized into four diagnostic tiers: (1) Species-positive (≥1 valid species-specific target); (2) CaPV-positive/undetermined (conserved P1–P4 pass, but ultra-long P5–P7 fall below threshold due to low viral load or target decay); (3) Negative (no valid targets); or (4) Inconclusive (<10,000 total effective reads). For future clinical deployment, this decision system will transition to a three-tiered defense framework: multi-target voting (requiring ≥2 positive specific targets) to suppress single-target noise, mandatory ultra-long amplicon positivity for species confirmation, and ratio-based dynamic normalization (species-specific to total CaPV reads) to offset depth fluctuations. Given the proof-of-concept design with limited biological replicates (*n* = 1 per gradient), statistical comparisons across cohorts remain descriptive, with target regions within single technical runs serving as observational units.

To comprehensively evaluate and compare the cross-platform analytical performance, seven quantitative metrics were systematically defined: (1) Time-Normalized Throughput: total gigabases generated divided by the total sequencing time (4 h for ONT, 36 h for MGI); (2) Mapped Reads per Hour: absolute target-mapped reads generated per hour; (3) Target Capture Efficiency: the proportion of target-mapped reads relative to total effective reads; (4) Species ID Specificity: the proportion of strictly LSDV-specific reads out of all CaPV-mapped reads; (5) Background Purity: calculated as 1 minus the proportion of unmapped host or environmental background reads; (6) Data Output Stability: the inverse of the coefficient of variation (CV) in sequencing depth across all targets; and (7) Effective Data Output Efficiency: the percentage of raw reads that successfully passed quality and length filtering.

## 3 Results

### 3.1 Targeted primer library design and *in vitro* amplification validation

**Figure 1:**
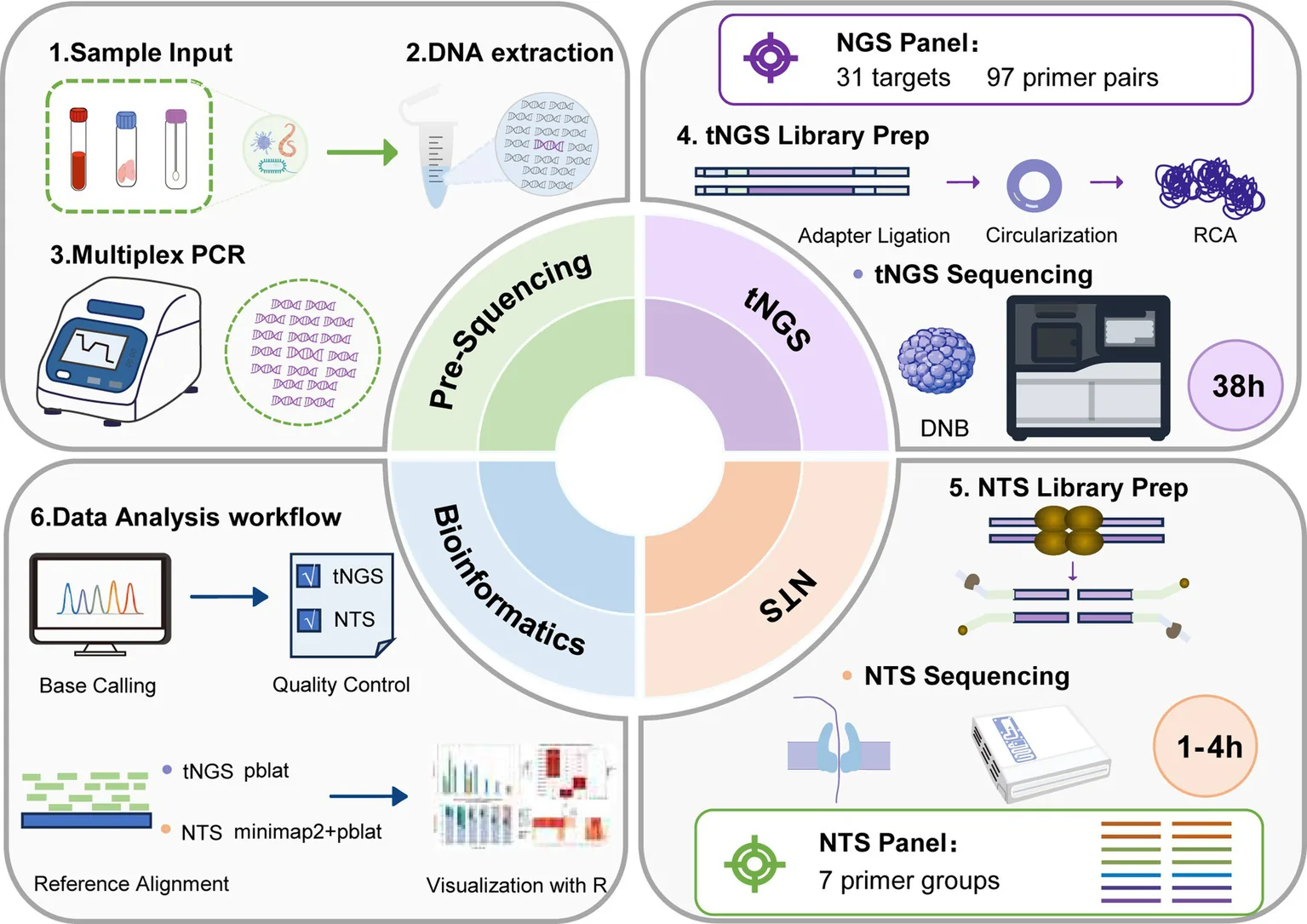
Schematic overview of the targeted sequencing workflows for LSDV detection using short-read targeted sequencing and NTS platforms.

Through whole-genome MSA and rigorous parameter screening, this study successfully constructed targeted amplification panels catering to different application scenarios and established in vitro amplification systems (Figure 1). In the short-read amplicon system aimed at laboratory screening, 31 functional gene regions with significant sequence differences and clear annotations were prioritized. A total of 97 pairs of specific short primers were successfully designed and synthesized, with target amplicon lengths distributed in the 100–200 bp range (primer sequences in Supplementary Table S1) and internal nucleic acid homologies strictly below 98%, laying the foundation for highly sensitive species-level identification. In the NTS long amplicon system designed for frontline rapid identification, an unbiased screening strategy using PrimalScheme was adopted. Combined with a low homology threshold of <97%, candidate long-fragment target regions rich in species-specific SNPs and Indels were successfully retained. These targets were scientifically divided into 7 multiplex reaction groups, covering the design gradients from 500 bp to 2000 bp (primer sequences in Supplementary Table S2).

**Figure 2:**
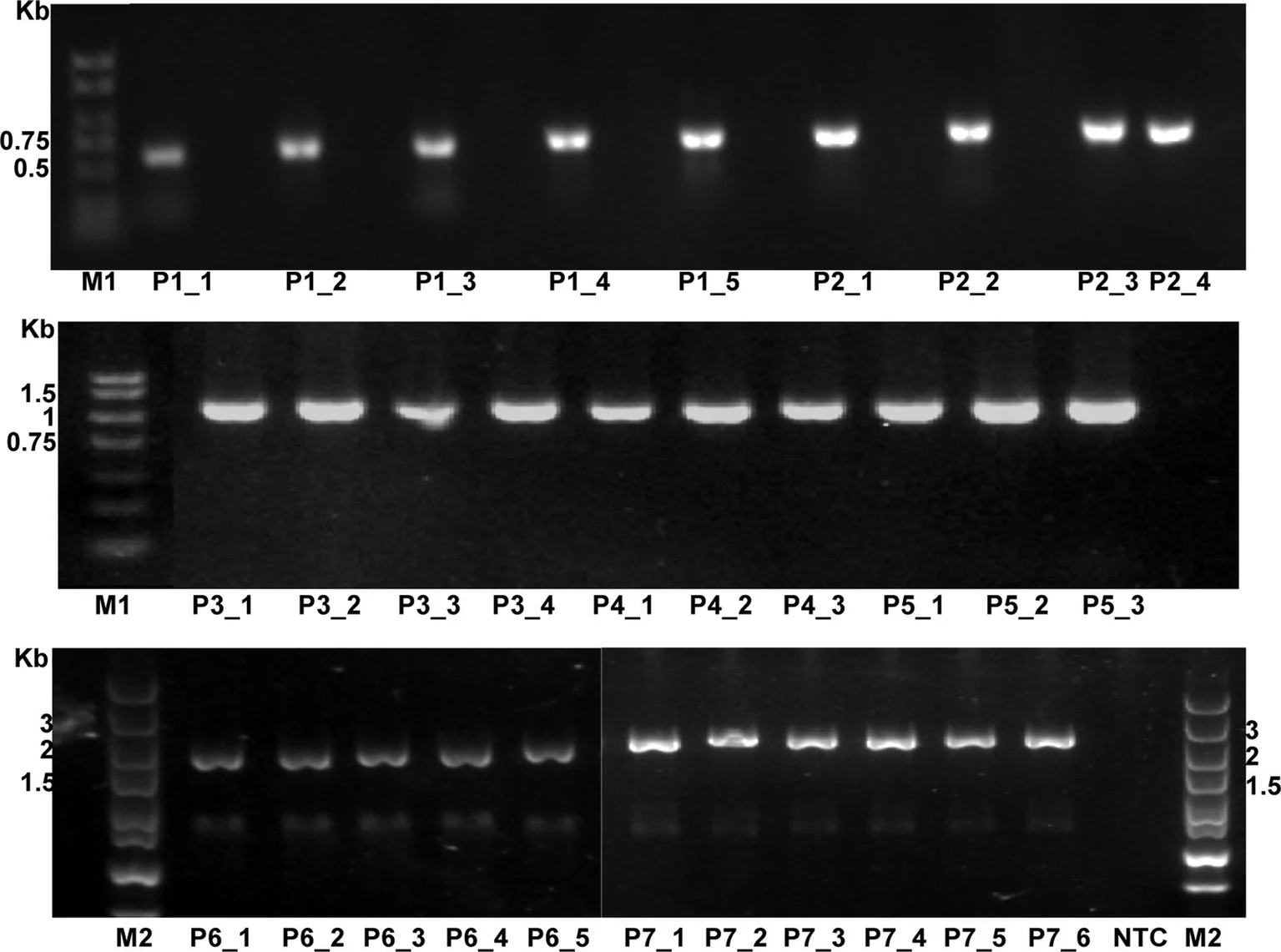
Agarose gel electrophoresis of singleplex PCR products for long-fragment targets. The selected core long primers were validated using purified LSDV templates. Lanes designated as Pn_m correspond to the m-th primer pair from the n-th primer group (e.g., P1_1-P1_5 are primer pairs from group 1). M1: DNA marker 2000; M2: DNA marker 5,000; NTC: No-template control.

To verify the amplification efficacy of the designed primers, in vitro experimental validations were conducted. Singleplex PCR and agarose gel electrophoresis results showed that the selected core long primers exhibited high specificity under the tested conditions in LSDV (Figure 2), GTPV (Supplementary Figure S1), and SPPV (Supplementary Figure S2) templates, stably generating single target bands with no non-specific amplification observed in the negative controls. These validated primers were directly formulated into long-fragment multiplex reaction groups, successfully establishing a multiplex system ready for downstream long-read sequencing library construction.

### 3.2 Identification and analytical performance evaluation of LSDV based on short-read sequencing

The multiplex panel prepared from 97 primer pairs was tested on the sequencing platform with the following sample setups: three concentration gradients of LSDV samples spanning an order of magnitude from 10^1^ to 10^3^ copies/μL (corresponding to precise baseline values of 5.26 × 10^3^, 5.26 × 10^2^, and 5.26 × 10^1^ copies/μL, one each), four replicates of GTPV samples, and an nuclease-free water negative control.

#### 3.2.1 Overall yield and coverage efficiency of the targeted sequencing system

**Figure 3:**
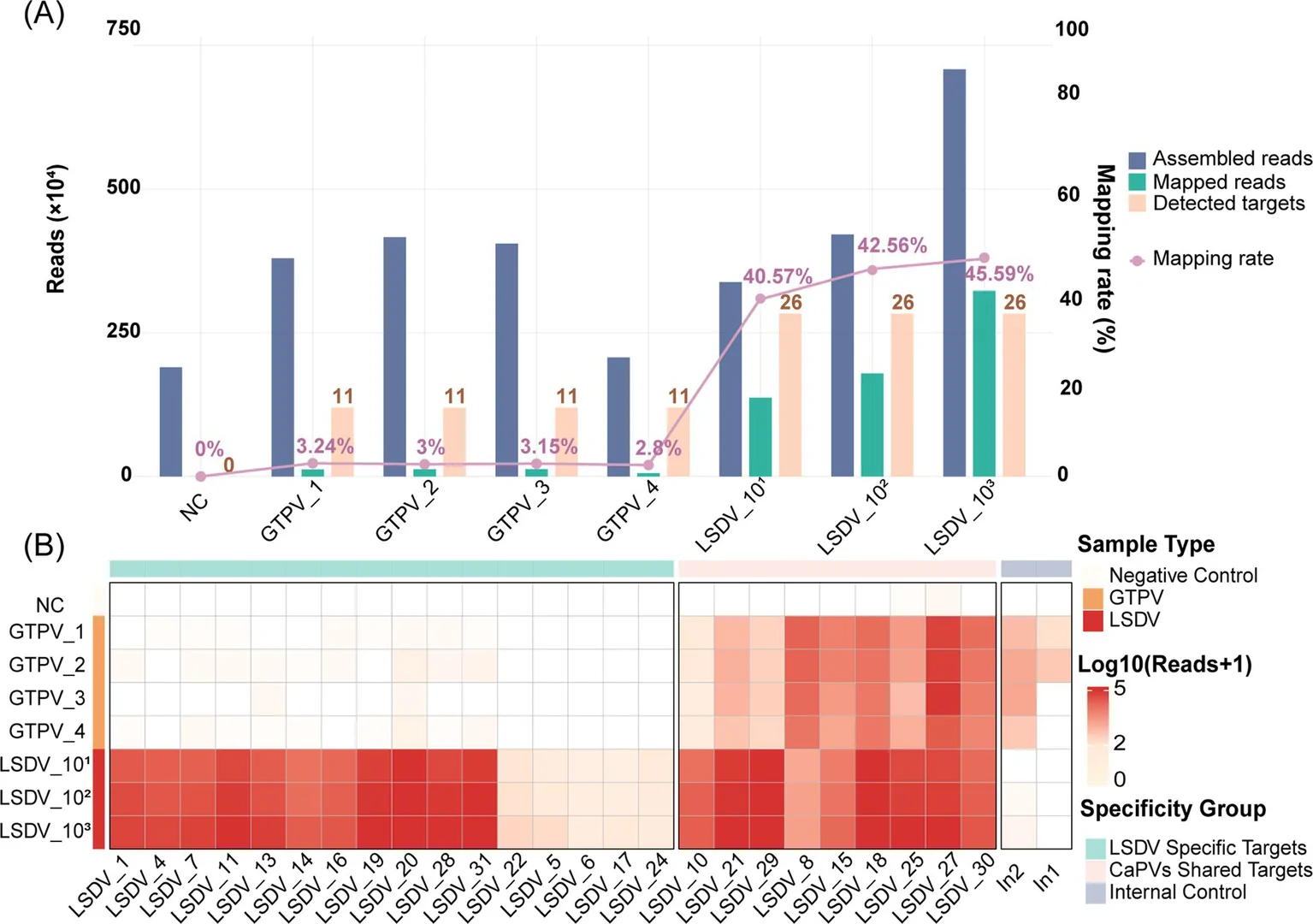
Analytical performance and target specificity of the targeted sequencing panel for LSDV detection. **(A)** Quantitative assessment of sequencing yields and mapping efficiencies. Bar charts display the number of assembled reads (dark blue), mapped reads (green), and detected targets (light orange) across negative control, Goatpox virus, and Lumpy Skin Disease Virus at varying concentrations (LSDV_0.1 to LSDV_10). The pink line indicates the overall mapping rate. **(B)** Heatmap illustrating the detection profile and specificity of individual targets within the panel.

The sequencing data statistics demonstrated that the detection system possessed sufficient and stable data acquisition capabilities. Excluding the negative control, which generated no effective data, the remaining 8 actual samples produced a total of approximately 62.26 M paired-end reads. The raw data volume for different samples ranged from 4.49 M (GTPV_4) to 15.35 M (LSDV_10). After quality control and sequence assembly, the final assembled reads for each sample ranged between 2.07 M and 7.08 M (Figure 3A). In high-concentration samples (10^3^ copies/μL), most target regions achieved high amplification saturation. Among them, the primer pair targeting the LSDV_20 region was the most efficient, yielding 0.76 M reads (Supplementary Table S3). Even in the test group where the total nucleic acid concentration was as low as 10^1^ copies/μL, the sample still obtained a stable total read output of 3.4 M, proving that the panel maintains reliable detection capabilities under low template conditions.

#### 3.2.2 Species-specific identification capability analysis

To verify the precise identification capability of this short-read targeted sequencing system for highly homologous CaPVs, this study conducted a systematic analysis combining macroscopic mapping rates and microscopic target abundances.

Under the strict alignment thresholds of 100% sequence identity and assembled read length > 120 bp, the effective mapping rate of LSDV samples remained stable at a high level (42.91% ± 2.53%, range, 40.57–45.59%), with 25 effective targets detected. In contrast, the mapping rates of the non-target GTPV samples experienced a substantial descriptive decrease (3.05% ± 0.19%, range, 2.80–3.24%), and the number of targets declined to 11 (Figure 3A). This result demonstrates from a statistical perspective that a strict alignment filtering strategy can eliminate the cross-signals of homologous viruses, thereby safeguarding the specificity of pathogen identification in complex samples.

**Figure 4:**
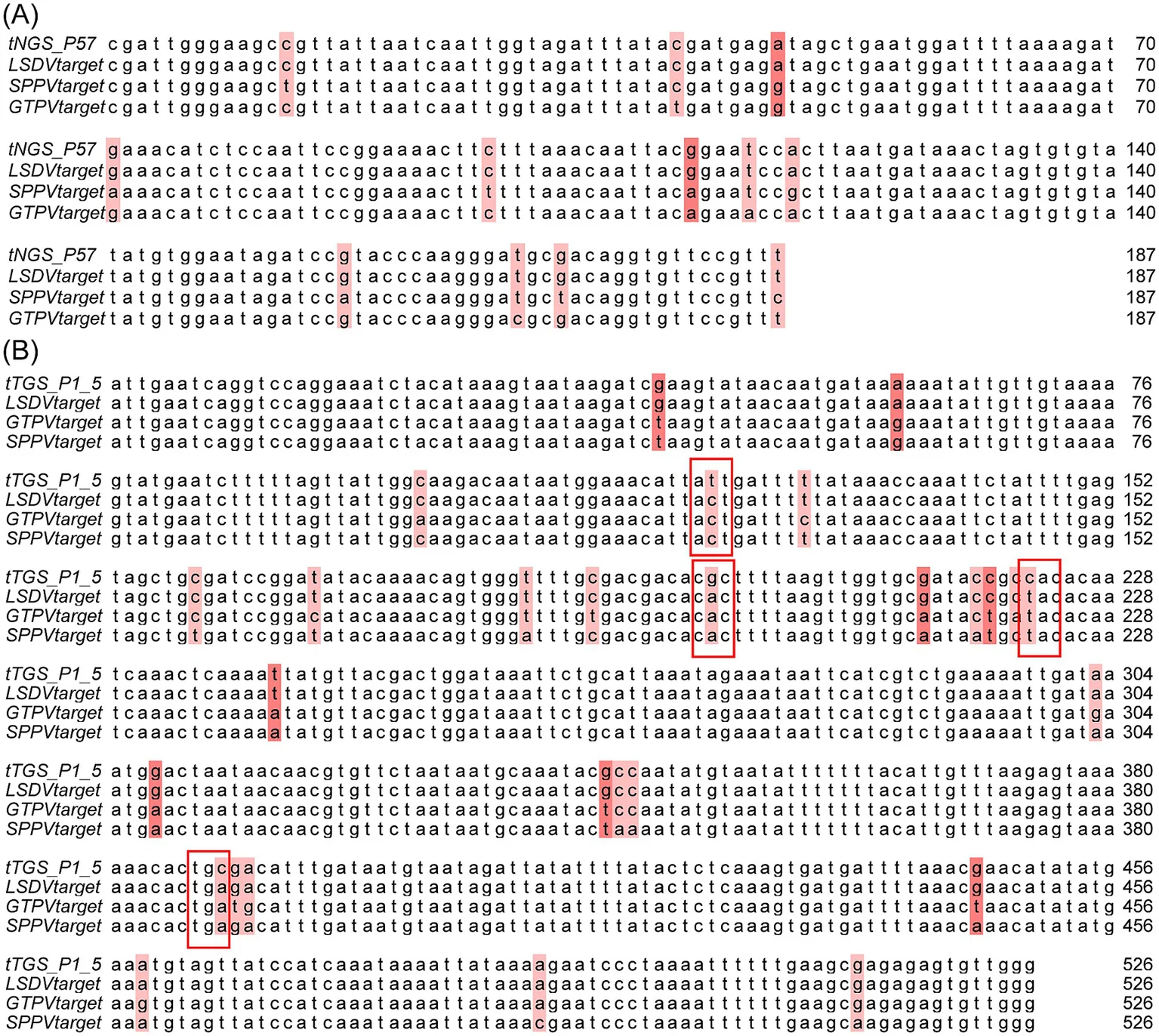
Sequence alignments demonstrating species-specific SNPs and novel mutations captured by targeted sequencing. **(A)** The 187 bp short-read targeted sequencing amplicon (LSDV_20_p27). **(B)** The 526 bp TGS long amplicon (P1_5). Annotations: Dark red shading, strict species-specific SNPs (LSDV differs from both GTPV and SPPV); light red shading, partial differentiating SNPs (LSDV differs from either GTPV or SPPV); red dashed boxes, novel sample-specific mutations distinct from all three reference genomes.

**Figure 5:**
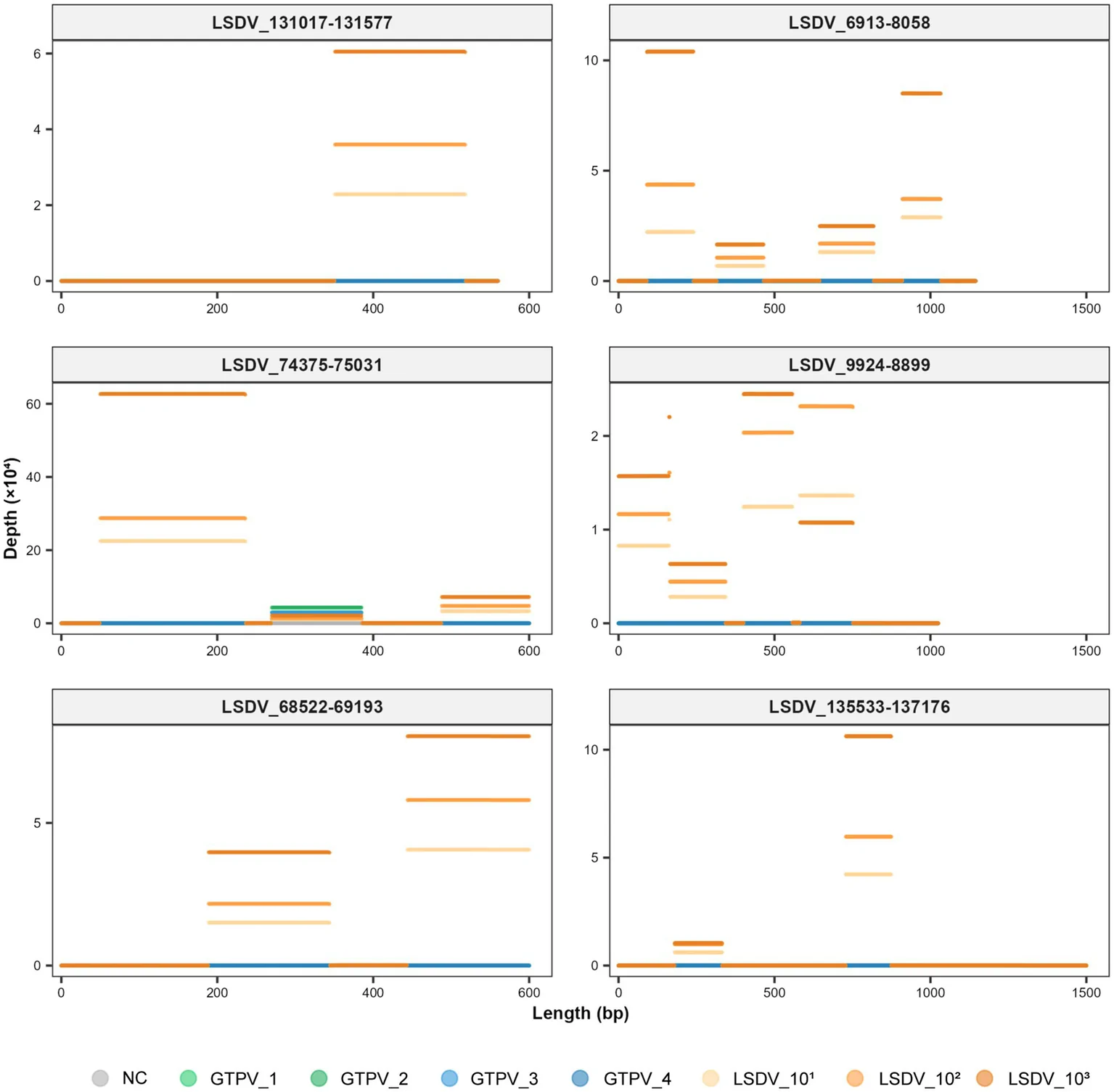
Sequencing depth distribution of the short-read targeted sequencing panel. Faceted line plots demonstrate the mapping depth across multiple specific genomic targets for different sample types and concentrations on the short-read targeted sequencing platform.

Further analysis (Figure 3B) examined the precise enrichment patterns of the 25 targets in LSDV and GTPV samples. The data showed that among the total 25 targets, 16 were identified as LSDV-specific discriminatory targets. Within this subset, 11 core target regions (including LSDV_1, LSDV_4, and LSDV_7) demonstrated exceptionally high species specificity and outstanding enrichment efficiency. In LSDV samples, these targets generated high abundances of effective reads (average reads: 102,806; range: 11,564-758,608). Conversely, in GTPV samples, the effective mapping reads in the same regions experienced an order-of-magnitude exponential decay, essentially lingering at background noise levels (average reads: 2.02 ± 3.37; range: 0–17), demonstrating a clear and unambiguous numerical separation between the target and non-target templates. Taking the LSDV_20 target as an example, in identically concentrated LSDV and GTPV samples, its effective reads decreased from 374,316 to 17, a level comparable to the negative control. Multiple sequence alignment of the specific 187 bp amplicon (generated by the LSDV_20_p27 primer pair targeting a sub-region of the LSDV_20 locus) against the reference genomes clearly illustrates the molecular basis for this specificity. The presence of distinct single nucleotide polymorphisms (SNPs) within this short amplification window effectively prevents non-target amplification and bioinformatic cross-mapping (Figure 4A). Sequencing depth distribution corroborated this specificity: LSDV samples presented continuous high-depth coverage peaks in specific target regions (up to 627,110×, with all 11 targets exceeding 1,000 × in depth), while GTPV samples essentially remained near the baseline level (Figure 5). Under the strict alignment threshold of 100% sequence identity, non-target viruses were effectively filtered out due to specific single nucleotide polymorphism (SNP) mismatches within the amplicons, thereby achieving precise locking onto LSDV.

Notably, empirical validation revealed that the final panel encompasses two distinct functional categories of targets. While the 16 specific targets (e.g., LSDV_1, LSDV_4) primarily amplified the target virus to enable precise species-level differentiation, the 9 targets in the “CaPVs Shared Targets” group demonstrated stable read enrichment across both LSDV and GTPV. This observation leads us to speculate that the primer-binding regions of these 9 targets are highly conserved within the Capripoxvirus genus, allowing them to serve as conserved markers for preliminary genus-level screening. However, because the genomic regions covered by these 100–200 bp short amplicons are limited, their internal sequences do not encompass sufficient inter-species variations to distinguish LSDV from GTPV. Therefore, within this multiplex system, these two target categories fulfill complementary diagnostic roles: the shared targets provide broad-spectrum preliminary amplification, while the specific targets ensure exact species identification.

#### 3.2.3 Evaluation of detection capability across varying input concentrations

To evaluate the detection capability of the panel for different starting template amounts, gradient dilution sequencing was performed on LSDV samples at three concentrations: 10^3^, 10^2^, and 10^1^ copies/μL. The results indicated that as the starting nucleic acid concentration decreased, the sequencing yield and coverage depth of each target showed an expected gradient decline, but the system maintained reliable detection capability at low concentrations. In the high-concentration (10^3^ copies/μL) sample, all specific target regions exhibited high and continuous depth coverage, with most targets reaching amplification saturation. When the concentration dropped to 10^1^ copies/μL, the depth in all regions decreased uniformly, with target reads being approximately 1/3 to 1/5 of the high-concentration group; however, all key targets were still clearly detected, and the signal intensity far exceeded the detection threshold. Even at the lowest test concentration (10^1^ copies/μL), the system still acquired 3.4 M effective reads, and the 11 core specific targets (LSDV_1, LSDV_4, LSDV_7, etc.) all maintained clear effective signals, showing a significant difference from the negative control with no missed detections (Figures 3A, 5). These results demonstrate that the panel can reliably enrich LSDV targets across a broad concentration range spanning two orders of magnitude, exhibiting excellent template amount tolerance. Specifically, the viral targets remained detectable at the lowest total nucleic acid input of 10^1^ copies/μL tested in this study.

The short-read amplicon panel not only possesses broad-spectrum initial screening capabilities at low concentrations but also demonstrates precise identification efficacy based on specific SNPs, making it a highly reliable detection strategy for laboratory environments. However, the 100–200 bp short amplicons possess an inherent physical limitation: the number of discriminative SNP loci captured within a single short-read sequence is strictly limited. Relying on a minimal number of SNPs for species-level identification makes the assay vulnerable; if a natural mutation occurs at these specific loci during viral evolution, it could lead to identification inaccuracies. Furthermore, short sequences cannot directly span and resolve large-scale complex structural variations (Indels). These limitations prominently highlight the necessity of introducing a NTS long-fragment identification system. By capturing extended sequences, NTS provides richer and more robust variation information, thereby enabling complex variation characterization and rapid on-site application.

### 3.3 Multiplex targeted amplification efficacy and cross-platform comparative evaluation based on NTS

#### 3.3.1 Sequencing yield and species specificity analysis of long amplicon primer sets

To break through the limitations of short-read sequencing in resolving large-scale structural variations and to develop an integrated detection scheme suitable for on-site use, we designed a series of multiplex PCR primer sets with amplicon lengths ranging from 500 bp to 2,300 bp and performed sequencing analysis on LSDV, GTPV, and SPPV samples using the Oxford Nanopore Technologies (ONT) platform.

**Figure 6:**
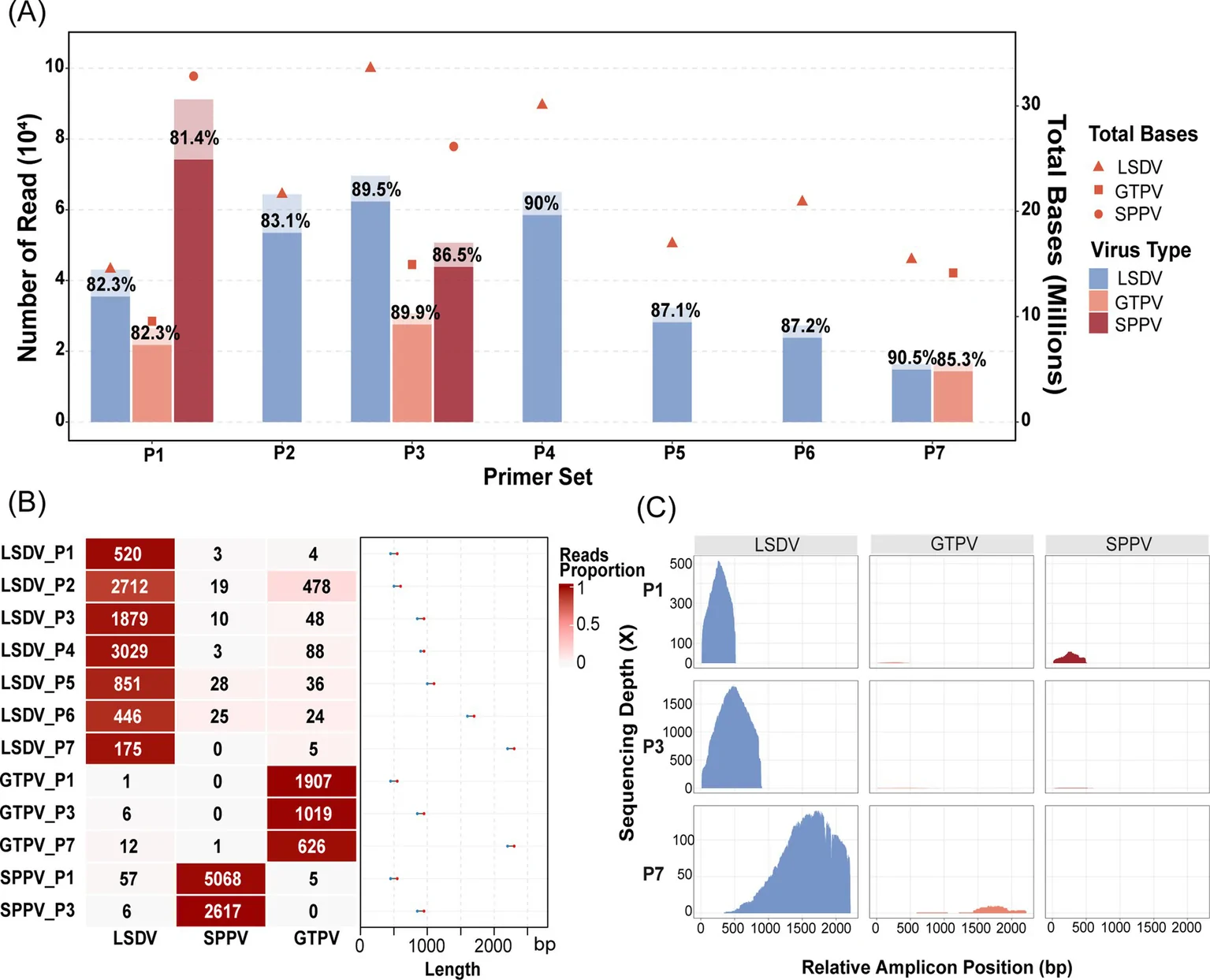
Amplification efficiency and specificity of the Nanopore-Targeted Sequencing (NTS) panel. **(A)** Read distribution and total sequenced bases across seven specific primer sets on the NTS platform. **(B)** Cross-reactivity matrix for species-specific primer combinations. **(C)** Sequencing depth distribution mapping across relative amplicon positions for the target viruses.

Statistical analysis of the sequencing data for different long-fragment primer sets in LSDV, GTPV, and SPPV samples revealed that all loaded samples successfully achieved high-abundance sequencing (Figure 6A). Each primer group produced high-abundance reads on the order of tens to hundreds of thousands, as well as millions of total bases, demonstrating robust large-volume data acquisition capabilities. The percentage values in the figure represent the effective mapping rate of the sequencing products for each group aligned to the LSDV reference genome via the Minimap algorithm. The data indicated that when LSDV target samples were input, the 7 primer products demonstrated stable, high mapping rates against their own reference sequences (e.g., P3 group 89.5%, P5 group 89.9%, P7 group 90.5%). Meanwhile, when GTPV and SPPV samples were input, the mapping rates of their corresponding sequencing products against the LSDV reference genome also exhibited high levels (e.g., up to 81.4% for SPPV samples and 86.5% for GTPV samples). This phenomenon directly and objectively reflects the biological characteristic of high genomic sequence identity among species within the Capripoxvirus genus.

To accurately evaluate the species identification capability of the primers, we comprehensively analyzed the targeted reads distribution matrix (Figure 6B) alongside the sequencing depth distribution (Figure 6C). The results demonstrated a clear interplay among amplicon length, amplification efficiency, and specificity. For shorter amplicons (P1-P4, 500–900 bp), the system demonstrated high amplification efficiency, yielding thousands of targeted reads (e.g., >1800–3,000 for LSDV) and presenting high-depth coverage peaks (Figure 6C). Importantly, as shown in Figure 4B, a representative 526 bp amplicon (generated by the P1-5 primer pair from the P1 group) captures a significantly higher density of species-specific SNPs compared to the short-read targets. This extended variation profiling allows for precise species differentiation even within relatively conserved genomic regions. However, because these shorter fragments cover conserved homologous sequences, they generated minor cross-reads in non-target samples (e.g., 478 cross-reads for LSDV_P2 in GTPV), which manifested as weak cross-coverage signals in the depth distribution.

As amplicon length extended into the ultra-long range (P6-P7, >1,600 bp), total read yields and absolute sequencing depth decreased due to multiplex PCR efficiency limits. Nevertheless, these ultra-long fragments demonstrated high species specificity by targeting high-density differentiated regions. For instance, the mismatched reads for LSDV_P7 (> 2,200 bp) decreased to 0 in SPPV and 5 in GTPV, while GTPV_P7 also exhibited minimal cross-reactivity (12 and 1 in LSDV and SPPV, respectively). Figure 6C visually corroborates this, showing that non-target cross-signals were thoroughly suppressed to near background noise levels. This integrated analysis validates our methodological design: short amplicons are optimal for high-depth genus-level screening, whereas ultra-long amplicons are essential for precise species-level identification by eliminating homologous interference.

#### 3.3.2 Cross-platform applicability of the NTS targeted system

To evaluate the adaptability and robustness of the long-fragment targeted multiplex primer sets developed in this study across different sequencing platforms, the same batch of amplification products was sequenced in parallel on the ONT platform and the MGI platform. The results showed that, despite inherent technical differences between the two platforms in sequencing time (4 h for ONT, 36 h for MGI) and data output characteristics, this primer system demonstrated stable targeted amplification performance on both platforms.

**Figure 7:**
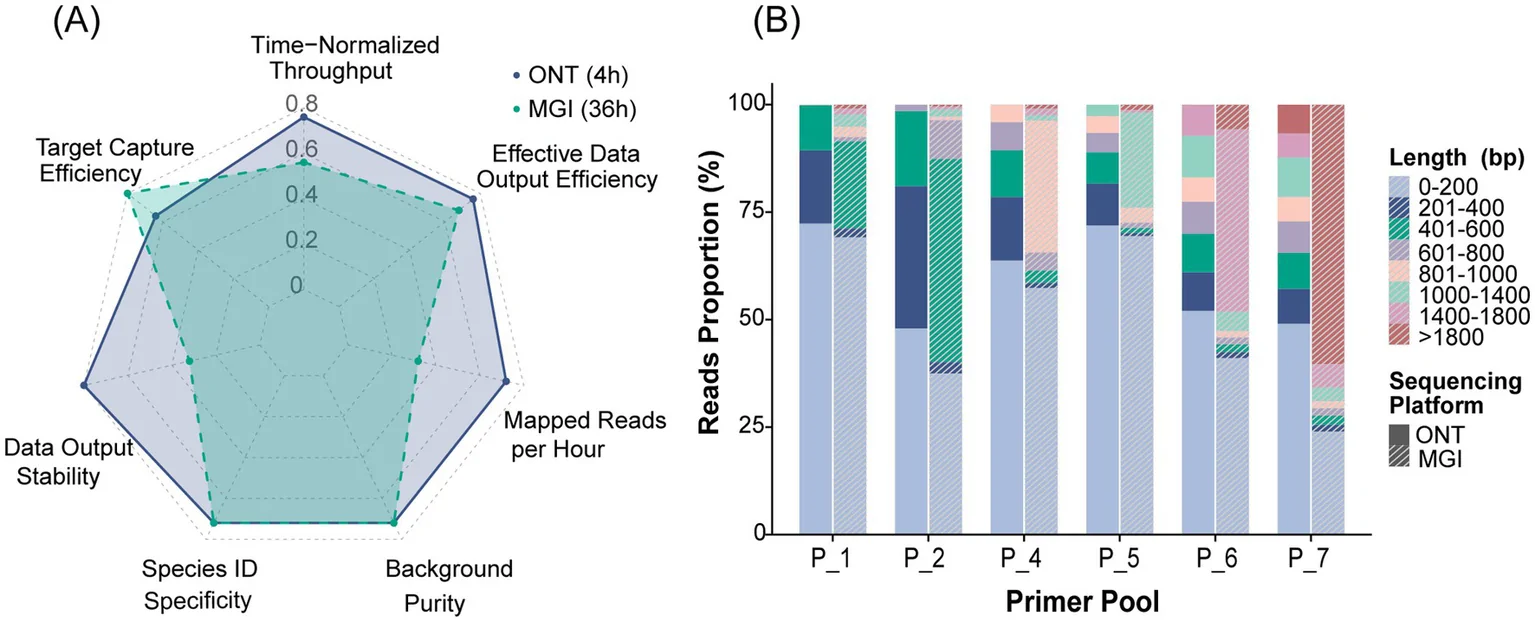
Comprehensive analytical performance comparison between the ONT and MGI platforms. The evaluation was conducted using the same batch of purified long-fragment multiplex PCR products from an LSDV-positive sample (*n* = 1 technical run per platform). **(A)** Radar chart summarizing key performance metrics. Raw metric values were min-max normalized to a 0–1 scale to enable cross-dimensional comparison. Detailed quantitative definitions and mathematical rationales for the seven performance metrics are strictly defined in Section 2.6. **(B)** Stacked bar charts illustrating the read length distribution across six representative primer pools. Values represent the proportion (%) of effective mapped reads falling within distinct length bins (from 0–200 bp to >1800 bp) for the ONT and MGI platforms.

The two platforms demonstrated high consistency in core identification capabilities but exhibited distinct performance profiles in sequencing efficiency and output characteristics (Figure 7A). The ONT platform exhibited marked advantages in three time and data acquisition dimensions: “Time-Normalized Throughput,” “Mapped Reads per Hour,” and “Data Output Stability.” Comparatively, the MGI platform held a slight edge in the “Target Capture Efficiency” metric. On the two core metrics determining pathogen typing accuracy, “Species ID Specificity” and “Background Purity,” both platforms achieved the same high level of efficacy.

Regarding the amplicon fragment length distribution characteristics of the output data from both platforms, this study conducted interval statistics for six primer products: Primer_1,2,4,5,6 and 7 (Figure 7B). The results showed that, facing the same batch of multiplex PCR products, the length bins ultimately captured by the ONT and MGI platforms exhibited significant differences. Specifically, in the Primer_6 and Primer_7 groups containing longer expected amplicons, the MGI platform retained a high proportion of sequences in the long-fragment intervals of >1,400 bp and >1800 bp. Conversely, the proportion of long-fragment intervals on the ONT platform decreased relatively, with the overall distribution shifting toward the medium-short fragment intervals. The main reason for this discrepancy in sequencing length distribution lies in the different library construction methods: the ONT platform utilized a rapid library construction method based on transposases. The transposase complex randomly cleaves the original long-fragment amplification products while inserting sequencing adapters, thus physically altering the actual fragment sizes loaded onto the machine.

### 3.4 Evaluation of the application potential of the ONT-based workflow in frontline scenarios

The long-read multiplex PCR detection scheme based on the ONT platform established in this study highly aligns with the urgent need for rapid on-site diagnostic technologies in frontline scenarios such as farms, slaughterhouses, and customs quarantine stations (Eltom et al., 2021). Unlike infrastructure-dependent short-read sequencing platforms, ONT sequencers (e.g., the MinION) are highly portable (weighing < 500 grams), feature simple library construction workflows, and do not require complex laboratory environments (Ji et al., 2024). To formally evaluate the practical feasibility of this ONT-based workflow for frontline deployment, we systematically monitored the execution time of each step. The entire workflow, from crude sample to an initial diagnostic early warning, can be accomplished within approximately 4 h, while the comprehensive full-depth sequencing performed for this study’s statistical analysis was completed in under 8 h. The detailed time breakdown and operational parameters demonstrated in this study are summarized in Table 1.

**Table 1:** Time breakdown and operational parameters of the portable ONT-based targeted sequencing workflow for frontline deployment.

Step	Time required	Operational parameters and details
DNA Extraction	30 min	Nucleic acids were extracted using a commercially available portable spin-column rapid extraction kit, requiring only a benchtop mini-centrifuge and minimal pipetting infrastructure.
Multiplex PCR Amplification	50–90 min	Amplification was performed using a fast thermal cycling program on a compact, field-deployable thermocycler, successfully yielding sufficient target amplicons.
Amplicons Purification	15 min	To maintain field-deployability, post-PCR products were purified using Agencourt AMPure XP magnetic beads with a portable magnetic rack (15 min hands-on time).
ONT Library Preparation	50 min	Utilizing the ONT rapid barcoding kit, the transposase-based cleavage and indexing wrapper were accomplished with optimized isothermal incubations.
Sequencing and Real-time Early Warning	Initial Detection: 60 min	The library was loaded onto an ONT MinION portable sequencer. Sufficient species-specific reads (> 50 absolute reads) stably accumulated within the first 60 min, allowing an immediate early warning for quarantine interception. The run was maintained for 4 h to ensure robust sequencing depths and comprehensive datasets for analysis.
	Full Acquisition: 4 h	
Bioinformatic Analysis	20 min	The computational pipeline (including basecalling, quality control, alignment, and classification) processed the 4-h dataset to generate the final diagnostic report.

### 3.5 Evaluation of the targeted sequencing panel in clinical specimens and complex matrices

To evaluate the diagnostic performance of the established targeted sequencing protocol, three primer sets representing distinct amplicon lengths (P1, P3, and P7) were evaluated using a combination of spiked gradient samples and authentic clinical specimens. The evaluated sample cohort comprised true positive clinical specimens, true negative blood/tissue matrices, and a standard LSDV serial dilution gradient (10^1^, 10^2^, and 10^3^ copies/μL) spiked into negative blood/tissue nucleic acid extracts.

**Figure 8:**
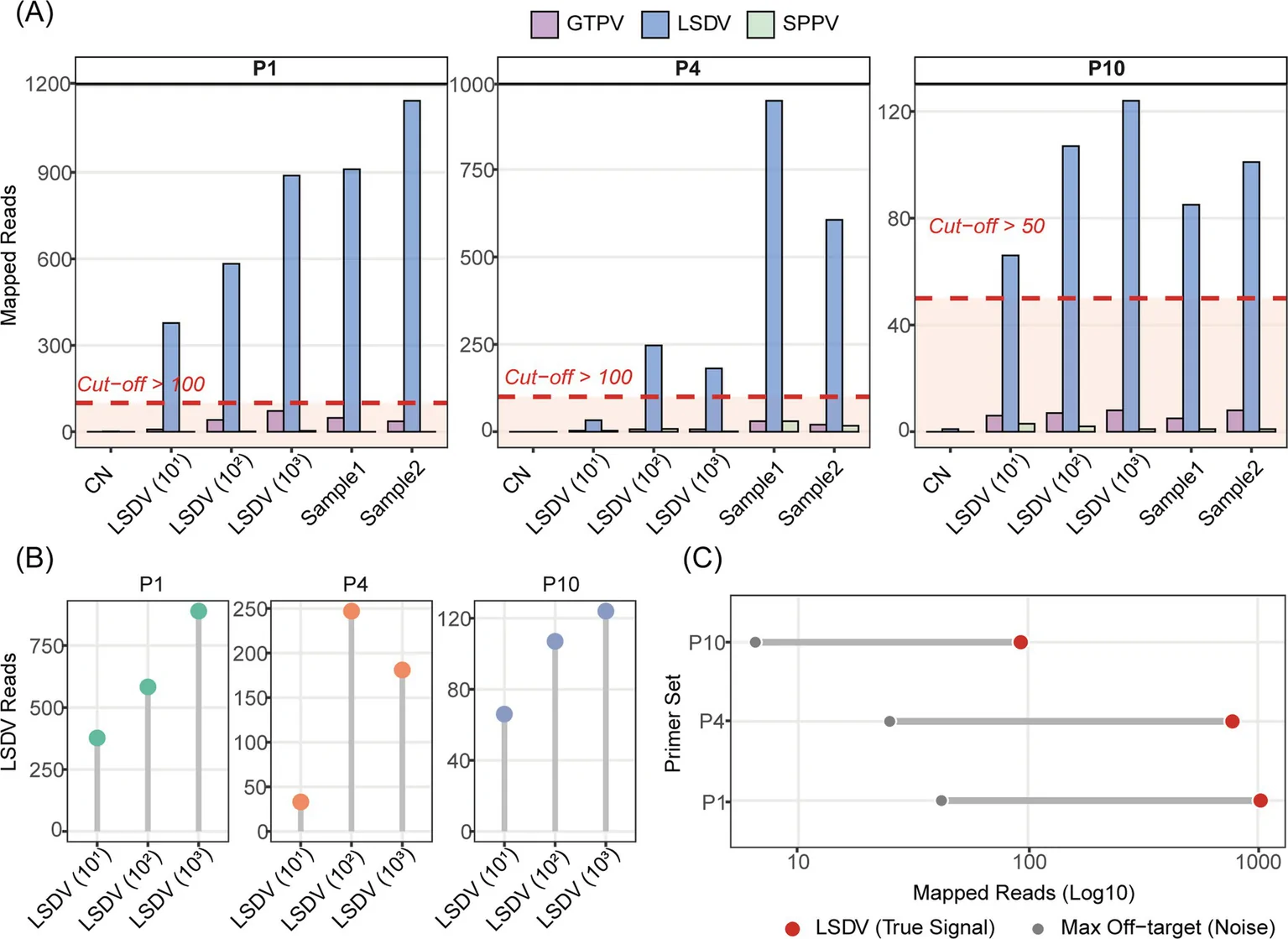
Analytical performance of the targeted sequencing assay. **(A)** Target read counts across representative primer sets (P1, P3, and P7) in clinical positives (Sample 1: 4.27 × 10^4^ copies/μL; Sample 2: 1.49 × 10^4^ copies/μL), LSDV dilutions spiked into negative blood/tissue extracts (10^1^–10^3^ copies/μL), and clinical negative controls (CN). Dashed lines and shaded areas indicate diagnostic thresholds (>100 reads for P1/P3; >50 reads for P7). **(B)** Target reads distribution across the LSDV spiked gradient (10^1^–10^3^ copies/μL). **(C)** Comparison of mapped target reads (red) and maximum off-target reads (grey) on a Log_10_ scale.

As shown in Figure 8A, the targeted assay exhibited target enrichment capabilities, though detection sensitivity varied across primer configurations. Primer sets P1 and P7 demonstrated higher analytical sensitivity than P3; across all positive conditions, including the 10^1^ copies/μL concentration and clinical positive samples, the mapped LSDV read counts for P1 and P7 consistently exceeded their respective diagnostic thresholds (Cut-off > 100 for P1; Cut-off > 50 for P7). In contrast, while primer set P3 successfully detected targets at 10^2^ and 10^3^ copies/μL as well as in clinical positive samples, its read count fell below the threshold at 10^1^ copies/μL, indicating a higher limit of detection compared to P1 and P7. Across all evaluated samples, cross-mapped read counts against highly homologous Capripoxvirus reference genomes (GTPV and SPPV) and read counts from clinical negative controls (CN) remained consistently below the established diagnostic thresholds (shaded region).

Following the stringent bioinformatic filtering pipeline, sequencing data were utilized to evaluate read distributions across varying viral loads. As shown in Figure 8B, targeted read counts correlated with viral input concentrations, exhibiting a proportional increase as the spiked concentration rose from 10^1^ to 10^3^ copies/μL. The marked reduction in target reads for P3 at 10^1^ copies/μL aligns with the limit of detection findings presented in Figure 8A.

To assess the ability of the method to discriminate against closely related pathogens, the relationship between genuine target signals and off-target reads was quantified (Figure 8C). On a logarithmic scale (Log_10_), a separation gap of 1.5 to 2.5 orders of magnitude was observed between mapped LSDV read counts and maximum off-target reads across all three primer sets. Under the stringency of the bioinformatic filtering pipeline, cross-mapping reads from homologous Capripoxviruses (GTPV and SPPV) were maintained at low levels. This logarithmic separation confirms that the workflow effectively differentiates genuine target signals from background off-target reads.

## 4 Discussion

The core innovation of this study lies in combining multiplex PCR targeted amplification with sequencing technologies to systematically resolve the technical challenges of precisely differentiating closely related species within the Capripoxvirus genus. In this study, the LSDV, GTPV, and SPPV nucleic acid samples were all unambiguously confirmed using the national standard qPCR assay, ensuring a reliable material basis for testing. Unlike conventional qPCR assays that rely on a single conserved target, our approach introduces a multi-target design at the whole-genome level, enabling the simultaneous enrichment and resolution of multiple specific locus intervals. Furthermore, compared to direct whole-genome sequencing (WGS) or metagenomic Next-generation sequencing (mNGS), this study effectively enriched target regions by designing specific primer libraries, overcoming the problems of insufficient detection sensitivity for low-viral-load samples and ambiguous bioinformatic alignment caused by highly homologous sequences.

While WGS remains the gold standard for comprehensive viral genomic characterization and broader phylogenetic analysis, its efficiency is limited when the diagnostic goal is strictly constrained to identifying closely related species (Charlier et al., 2026). LSDV, GTPV, and SPPV share over 96% genomic homology; thus, WGS indiscriminately covers the entire genomic sequence and generates massive amounts of redundant data. These highly homologous regions contribute nothing to species identification yet consume extensive sequencing depth and data output (Quainoo et al., 2017). In contrast, mNGS captures all nucleic acid molecules indiscriminately, which means target viral sequences are massively diluted by the host and environmental background. More critically, when mNGS is applied to highly homologous species, the generated short reads map ambiguously to the genomes of multiple related viruses. This multi-mapping issue creates severe bioinformatic cross-interference, making definitive species-level identification extremely difficult (Torres Montaguth et al., 2026).

The multiplex PCR targeted sequencing strategy employed in this study fundamentally circumvented the above defects. By screening low-homology regions as targets at the whole-genome level, sequencing data was guided to the genomic intervals with the most identifying value, avoiding data wastage on highly homologous redundant regions. Specifically, the short-read amplicon panel designed in this study increased the effective mapping rate of LSDV samples from the typically <1% level seen in mNGS to over 40% (Figure 3A) (Gu et al., 2019); in the NTS long amplicon panel, the number of cross-reads for ultra-long fragments (> 2,200 bp) in the GTPV sample was merely 5, and 0 in the SPPV sample, achieving a complete exclusion of closely related species (Figure 6B). This indicates that the targeted sequencing strategy directs every sequencing read to a meaningful identification region, elevating data utilization efficiency by orders of magnitude.

In the short-read amplicon panel, this study precisely screened 31 target regions with low homology between LSDV and GTPV/SPPV through full-genome MSA. After targeted enrichment, the 11 specific targets demonstrated exceptional specificity; based on the read distribution in non-target templates, the panel achieved an overall exclusion rate of > 98% against GTPV under strict alignment conditions, confirming the effectiveness of the targeted enrichment strategy. However, the amplification length of 100–200 bp determines that the number of capturable SNP loci is limited. When the diagnostic demand is further elevated to resolve even more closely related species, such as GTPV and SPPV, this inherently limited SNP density within short amplicons becomes a theoretical bottleneck. Notably, approximately 9 targets exhibited stably high read enrichment levels across both the tested LSDV and GTPV samples, suggesting that their primer binding regions are highly conserved between these two viruses. It is recommended that these conserved targets be further evaluated as candidate molecular markers for broad-spectrum screening within the genus. In addition, 5 targets showed low amplification efficiency, and subsequent studies will systematically optimize their sequences and amplification conditions.

The NTS long amplicon panel was designed to cover multiple length gradients, including 500–600 bp, 800–900 bp, 1,000–1,100 bp, 1,500 bp, and 2000 bp. By mandatorily setting an inter-species homology threshold of <97%, highly conserved regions were systematically excluded, effectively retaining differentiated regions rich in species-specific SNPs and Indels. The results indicate the validity of this design: ultra-long fragments (>2,200 bp) obtained high-depth coverage in LSDV samples, while the cross-reads in GTPV and SPPV samples decreased to 5 and 0, respectively. This outcome proves that longer amplicons can cover broader genomic intervals and accumulate far richer variation loci information, thus achieving a depth of identification unattainable by short fragments. It should be noted that while the rapid transposase-based library preparation (e.g., ONT SQK-RBK114) physically cleaves these ultra-long amplicons into slightly shorter sub-fragments (typically distributed between 800 and 1,500 bp), this does not compromise the fundamental advantage of NTS in our assay. Unlike the 100–200 bp short reads generated by short-read platforms, which require complex bioinformatic assembly and are prone to chimeric alignment errors in highly homologous genomes, these NTS sub-fragments still represent continuous, single-molecule sequences. They remain sufficiently long to stably span multiple co-segregating SNPs and structural variations (Indels) in a single continuous phase, preserving the absolute linkage information necessary for unambiguous species-level differentiation and mixed-infection resolution.

Despite these distinct methodological advantages, empirical data revealed technical aspects requiring systematic management. First, the amplification efficiency of ultra-long targets (e.g., P6, P7 groups) naturally decayed due to length constraints, whereas certain shorter NTS sets (e.g., P2 group) exhibited background cross-signals in non-target templates (yielding up to 478 cross-reads in GTPV). To robustly manage batch-to-batch sequencing depth variations and prevent false positives from high-titer non-target viruses, our framework integrates length-dependent cutoffs (≥100 reads for short amplicons; ≥50 for ultra-long amplicons) with strict bioinformatic filtering (98% identity over ≥250/400 bp). Crucially, the ultra-long amplicons intrinsically serve as run-specific specificity references; because they consistently yield near-zero cross-reads (≤5 reads) in non-target genomes, they instantly flag technical anomalies without requiring live parallel controls in every run batch. For full clinical deployment, this decision system will further evolve by adopting multi-target voting rules (requiring ≥2 positive targets), mandatory ultra-long target validation, and ratio-based dynamic normalization (species-specific to total CaPV reads) to completely neutralize run-to-run depth fluctuations.

The diversified targeted amplification panels established in this study can flexibly adapt to varying application scenarios. For laboratories equipped with high-throughput sequencing conditions, the short-read targeted sequencing panel enables rapid screening of large batches of samples. With 97 primer pairs covering 31 target regions, it stably detects LSDV over a broad concentration range of 5.26 × 10^3^ to 5.26 × 10^1^ copies/μL. Conversely, for rapid on-site detection scenarios, the ONT platform combined with the NTS long amplicon panel can generate reliable diagnostic results within a total workflow time of roughly 4 h. Notably, our empirical data demonstrated that even for the ultra-long P7 primer group (>2,200 bp), 57 effective species-specific reads were already captured at the 1-h sequencing mark. This early yield successfully reaches the diagnostic threshold, allowing for an immediate early warning. The sequencing run in this study was purposefully extended to 4 h strictly to acquire the robust, high-volume datasets required for comprehensive statistical analyses and depth distribution visualizations. Ultimately, with equipment weighing less than 500 g, this rapid NTS workflow boasts high practical feasibility for epidemic interception in grassroots quarantine scenarios with limited resources.

From a clinical validation perspective, this study provides a preliminary assessment of the assay’s performance using clinical samples and spiked matrix controls. By testing initial positive specimens and viral nucleic acid gradients (10^1^ to 10^3^ copies/μL) spiked into uninfected ruminant background matrices, we demonstrated the feasibility of the targeted sequencing workflow under simulated complex conditions. However, we acknowledge that the current evaluation is limited in scale. To fully establish its diagnostic utility and clinical reliability, future studies with larger, independent cohorts of real-world clinical samples are necessary. These subsequent validations will also incorporate a broader range of differential diagnostic targets, such as foot and mouth disease virus and parapoxviruses, to systematically evaluate potential cross reactions and establish definitive diagnostic cut-off for practical deployment.

It is worth emphasizing that the multiplex PCR targeted sequencing framework established in this study extends beyond lumpy skin disease. For pathogens that pose diagnostic challenges due to closely related species, our proposed strategy of combining whole-genome alignment for low-homology target screening, gradient-length amplicon design, and long-read sequencing for precise typing offers broad applicability. This integrated approach, coupling targeted enrichment with high-throughput sequencing, provides a valuable methodological blueprint for overcoming technical bottlenecks in distinguishing closely related pathogens. As NTS accuracy continues to improve and sequencing costs decrease, this framework is poised to become an essential diagnostic tool for the precise identification of lumpy skin disease virus and other closely related pathogens.

## Data Availability

The datasets presented in this study can be found in online repositories. The names of the repository/repositories and accession number(s) can be found in the article/Supplementary material.

## Glossary

AIV: Avian influenza virus
CaPVs: Capripoxvirus
CV: Coefficient of variation
ELISA: Enzyme-linked immunosorbent assay
FMDV: Foot-and-mouth disease virus
GTPV: Goatpox virus
Indels: Structural variants / Insertions and deletions
LAMP: Loop-mediated isothermal amplification
LOD: Limit of detection
LSD: Lumpy skin disease
LSDV: Lumpy skin disease virus
mNGS: metagenomic Next-generation sequencing
MSA: Multiple sequence alignment
NTC: No-template control
NTS: Nanopore targeted sequencing
ONT: Oxford Nanopore Technologies
ORFs: Open reading frames
PRRSV: Porcine reproductive and respiratory syndrome virus
qPCR: Quantitative real-time PCR
RPA: Recombinase polymerase amplification
SNP: Single nucleotide polymorphism
SPPV: Sheeppox virus
tNGS: Targeted next-generation sequencing
VNT: Virus neutralization test
WGS: Whole-genome sequencing
