# Supplementary material for: Development and evaluation of a multiplex PCR-based dual-platform targeted sequencing framework for precise differentiation of lumpy skin disease virus
Source: Front Microbiol. 2026 Aug 25;17:1916665. doi: 10.3389/fmicb.2026.1916665 (PMC13550171; doi:10.3389/fmicb.2026.1916665)
Supplement: Supplementary file 1 [file Data_Sheet_1.docx]

**Supplementary Figure S1**. Agarose gel electrophoresis of singleplex PCR products for long-fragment targets using GTPV templates. The selected core long primers were validated using purified GTPV templates. Lanes designated as Pn_m correspond to the m-th primer pair from the n-th primer group. M1: DNA marker 2000; M2: DNA marker 5000.

**Supplementary Figure S2.** Agarose gel electrophoresis of singleplex PCR products for long-fragment targets using SPPV templates. The selected core long primers were validated using purified SPPV templates. Lanes designated as Pn_m correspond to the m-th primer pair from the n-th primer group. M1: DNA marker 2000; M2: DNA marker 5000.
